# Supplementary material for: A comparative study on behavior, awareness and belief about cervical cancer among rural and urban women in Vietnam
Source: PLOS Glob Public Health. 2023 Jun 6;3(6):e0001817. doi: 10.1371/journal.pgph.0001817 (PMC10243615; doi:10.1371/journal.pgph.0001817)
Supplement: Attachment — Submitted filename: Vietnam CC review response 20230322 submitted.pdf [file pgph.0001817.s001.pdf]

SCHOOL OF PUBLIC HEALTH
EPIDEMIOLOGY
UNIVERSITY OF MICHIGAN

Department of Epidemiology ▪ Minh Tung Phung, PhD, MPH

March 22, 2023

Nnodimele Onuigbo Atulomah, PhD
Academic Editor
PLOS Global Public Health

Dear Dr. Atulomah,

Thank you for the opportunity to respond to the reviewers' and editor's comments. We appreciate the insightful comments and believe we have provided an improved manuscript. Point-by-point responses to the comments are provided below. We have provided the page and line numbers of our changes noted below based on the marked version of the manuscript. We have also reformatted the manuscript following the resubmission guide.

Journal Requirements:

1. Comment 1: Please review your reference list to ensure that it is complete and correct. If you have cited papers that have been retracted, please include the rationale for doing so in the manuscript text, or remove these references and replace them with relevant current references. Any changes to the reference list should be mentioned in the rebuttal letter that accompanies your revised manuscript. If you need to cite a retracted article, indicate the article's retracted status in the References list and also include a citation and full reference for the retraction notice.

   Response: We have reviewed the reference list and confirm that it is complete and correct. We have added a paragraph in Introduction based on Editor's comment #3 below and added one new reference. We have also renumbered the references based on their orders. However, we are not able to provide the tracked changes for renumbering the references using Microsoft Word.

2. Comment 2: Please send a completed 'Competing Interests' statement, including any COIs declared by your co-authors. If you have no competing interests to declare, please state "The authors have declared that no competing interests exist". Otherwise please declare all competing interests beginning with the statement "I have read the journal's policy and the authors of this manuscript have the following competing interests:"

   Response: We have revised the "Competing Interests" statement in the suggested format. Only one co-author has competing interests and we have reported them both in the manuscript page 19, lines 359-362 and in the submission system.

1415 Washington Heights
SPH Tower, Office
Ann Arbor, Michigan 48109-2029
phungmt@umich.edu

3. Comment 3: Please amend your detailed Financial Disclosure statement. This is published with the article. It must therefore be completed in full sentences and contain the exact wording you wish to be published.

a. State the initials, alongside each funding source, of each author to receive each grant.

b. State what role the funders took in the study. If the funders had no role in your study, please state: "The funders had no role in study design, data collection and analysis, decision to publish, or preparation of the manuscript."

c. If any authors received a salary from any of your funders, please state which authors and which funders.

If you did not receive any funding for this study, please simply state: "The authors received no specific funding for this work."

Response: We have revised the Financial Disclosure statement in the recommended format to report the funding sources. We have also added a statement that the funders had no role in this study in page 19 lines 350-358 as well as in the submission system.

4. Comment 4: Please ensure that Funding Information and Financial Disclosure Statement are matched.

Response: We have checked that Funding Information and Financial Disclosure Statement are matched.

5. Comment 5: We noticed that you used "data not shown and unpublished data" in the manuscript. We do not allow these references, as the PLOS data access policy requires that all data be either published with the manuscript or made available in a publicly accessible database. Please amend the supplementary material to include the referenced data or remove the references.

Response: We have removed the words "data not shown" and added the actual results in the manuscript, lines 230-234, pages 12-13.

Reviewer #1:
No comments.

Reviewer #2:

1. Comment 1: The authors should: (1) correct a typographical error on page 5 line 68 (Replace sexual with sexually)

Response: We are sorry for this typo. We have corrected it as "sexually transmitted disease".

2. Comment 2: (2) recompute the summary statistic on women who had undergone cervical cancer screening by the age of 35 years (see page 15 line 274).

Response: We believe we calculated the summary statistic correctly. As reported in lines 223-225 (page 12), there were 11/22 (=50%) rural women and 8/25 (=32%) urban women aged 30-35 who

1415 Washington Heights
SPH Tower, Office
Ann Arbor, Michigan 48109-2029
phungmt@umich.edu

reported ever being screened. When combining the rural and urban areas together, the percentage of women who reported ever being screened by the age of 35 years is ~40% (=19/47), which is correctly reported in line 290 page 15 (previously line 274).

Reviewer #3:

1. Comment 1: Title. I am suggesting a change in the title of the article for clear understanding of the study.
   The title could be change to "A comparative study on behavior, awareness and belief about cervical cancer among rural and urban women in Vietnam". This captured the results presented clearly.

   Response: We thank the reviewer for the suggestion. We have changed the title as suggested.

2. Comment 2: Abstract. Line 39-40: This statement should be written as "this study examined behavior, awareness and beliefs about cervical cancer screening among rural and urban women in this geographical region. The cross-sectional study should be removed, since it appeared in the methods.

   Response: We thank the reviewer for the suggestion. We have revised the sentence in lines 41-42 page 3 (previously lines 39-40) to make it less repetitive.

3. Comment 3: Line 54: it will be good to suggest approaches to increase the uptake of cervical cancer screening in that locality instead of stating that novel approaches are needed to increase uptake of cervical cancer. Since one of the study focus was to inform strategies to reduce cervical cancer burden in Southern Vietnam.

   Response: We thank the reviewer for this comment. Our findings of the concerns of physician-based screening methods being painful, awkward, embarrassing, and time consuming suggest that HPV self-sampling has great potential to improve screening uptake as this method does not require women to visit healthcare providers. Further, our findings that women lacked awareness of cervical cancer screening and that many would get screened if it was recommended by doctors and friends/family suggest the importance of programs that raise awareness and engage healthcare providers and network. We have revised the abstract to make it clear that HPV self-sampling, educational programs, and engaging healthcare providers and network are possible approaches to increase uptake of cervical cancer screening in this population in line 57, page 4.

4. Comment 4: Material and Methods. The authors did not specify how the sample size was calculated. The type of sampling technique used was not clearly stated.

   Response: This was an exploratory study and hence the sample size was driven by resource availability. It serves as a foundation for future studies on cervical cancer screening in this region. We used convenience sampling. In the Discussion, lines 322-329 page 17, we described the limitations of the study that we did not have access to population rosters and thus were unable to determine if participants were different from non-participants, and that the results may not be generalized to other areas. It is also possible that the lack of rural-urban differences observed in our

1415 Washington Heights
SPH Tower, Office
Ann Arbor, Michigan 48109-2029
phungmt@umich.edu

study was due to the small sample size, and we have added this as a limitation in lines 324-325, page 17.

5. Comment 5: Line 133: The authors stated that “participants watched short video that described cervical cancer screening before they filled the questionnaire”. I don’t think this is appropriate with the study design. I will like the authors to provide justification for during this.

   Response: For clarification, participants completed the sociodemographic and the cervical cancer awareness questionnaires before watching the video. The participants then watched the videos explaining different screening approaches prior to completing the questionnaire on cervical cancer screening uptake and beliefs about the different types of screening. We believe it is important for participants to watch videos to provide consistent information about screening as women might not know what cervical cancer screening is and whether they had had it. In fact, many participants told the researchers that they realized that they had previously had cervical cancer screening after watching the videos. Therefore, we believe that asking participants to watch short videos describing the cervical cancer screening methods reduced misclassification rather than introducing bias. We provided the justification in lines 285-286 (page 15) and lines 321-322 (page 17). We have further provided the justification early in the Methods lines 148-149 page 9.

6. Comment 6: Conclusion: Line 319: The authors concluded that they examined cervical cancer screening behavior, belief, and attitude. The results of the study did not cover attitude.

   Response: We thank the reviewer for pointing this out. We have revised the sentence in line 338 page 17 (previously line 319). We have also revised this through the manuscript.

Additional Editor Comments:

We thank the editor for taking a lot of time to provide comments on the manuscript. Broadly, we would say that while the Health Belief Model informed aspects of our questionnaire development, it was not the driving force behind the work. We can remove mention of the Health Belief Model from the methods if the editor would prefer for us to do that.

1. Comment 1: TITLE: This title provides limited information about the study and would greatly benefit from modifications considering that the theoretical underpinning of the problem phenomenon concerns in cervical cancer is screening in early diagnosis stage of secondary prevention, which is scientifically offered by the health belief model and public health principles of prevention and control.
   Strongly Suggested title: "Health-Belief constructs as predictors of cervical cancer screening among women in selected rural and urban communities of Southern Vietnam" With short title as; "Short title: Cervical cancer screening in rural and urban Southern Vietnam".
   Of course, "comprehensive assessment" need not be included in the title because the observational study is already an assessment of the problem phenomenon and seeks to elucidate the underpinning dynamics. Again the application of the theoretical framework has defined the rigor involved.

   Response: We thank the editor for the suggestion. We agree that the current title provides limited information about the study and we have revised it as requested by Reviewer #3. As explained in

1415 Washington Heights
SPH Tower, Office
Ann Arbor, Michigan 48109-2029
phungmt@umich.edu

more details in the response to Editor's comment #4 below, the health belief constructs are just one of the focuses of the study (besides screening uptake, structural barriers to screening, and awareness), and thus the Health Belief Model is not the guide of the entire study. We have revised the short title as suggested by the editor.

2. Comment 2: ABSTRACT: This needs to be appropriately written according to the required format for PLoS Global Public Health. All necessary information should be included Background, Objectives, Methods, Results and Findings with keywords.

   Response: When writing the abstract, we followed the guide in the journal website (https://journals.plos.org/globalpublichealth/s/submission-guidelines#loc-style-and-format), which states that: "*While the Abstract is conceptually divided into three sections (Background, Methodology/Principal Findings, and Conclusions/Significance), do not apply these distinct headings to the Abstract within the article file.*" We also checked the articles published in the most recent issue and their abstracts have no headings. Therefore, we have kept the abstract in the current format, but will be happy to change it if there has been a change in the journal requirements.

3. Comment 3: INTRODUCTION: All aspects of the introduction appear good showing the epidemiological principles but little of risk factors has been mentioned, public health principles of prevention and control with their respective modes of intervention are completely omitted but very essential to establish the underpinning public health scientific principles.

   Response: We thank the editor for the comment. As the editor suggested, we have added information on cervical cancer risk factors and HPV vaccination as a primary prevention strategy for cervical cancer. We have added a sentence in lines 65-67 (page 5) stating that the prevalence of cervical cancer risk factors such as premarital sex and early age at first sexual intercourse has been increasing to establish that cervical cancer is a public health priority. We have also added a paragraph in lines 76-81 (page 5) describing that HPV vaccination uptake is low in Southern Vietnam, in order to highlight the importance of cervical cancer screening.

4. Comment 4: Importantly, lines 81- 91 where the paper has introduced The Health Belief Model as theoretical framework underpinning health behaviour and health-seeking in health-risk condition typified by cancer of the cervix is weak and should be visible in the title because the construct provides the validity of the measurement in the study. The mention of this theoretical framework strengthens the scientific basis of the study, but it is also observed that this was casually mentioned and poorly structured to guide the entire study.

   Response: As stated in lines 92-94 page 6, this paper examined several factors associated with cervical cancer screening, including cervical cancer screening awareness, structural barriers to screening (such as cost or time traveling), and psychosocial barriers to screening. The health beliefs (or psychosocial barriers) are just one of the factors we assessed (besides awareness and structural barriers), and thus the Health Belief Model is not the emphasis of this study and is not the guide of the entire study. We appreciate the comment and are considering an additional manuscript that would focus more specifically on analyses related to The Health Belief Model, but it is outside the scope of this manuscript which is already very dense with novel findings.

1415 Washington Heights
SPH Tower, Office
Ann Arbor, Michigan 48109-2029
phungmt@umich.edu

5. Comment 5: Line 92: The objectives set for the study includes: evaluate level of knowledge about cancer of the cervix among women in urban-rural communities of southern Vietnam, and to inform strategies to reduce the cervical cancer in the region. But knowledge is not sufficient to trigger health-seeking behaviour, other antecedent variables in the theoretical framework are not set as course of action or specific objectives to guide the study.

   Response: We agree that more work is needed to evaluate different behavior change strategies. We are planning additional work based on our results.

6. Comment 6: What factors are referred to in line 94 which are not contained in the theoretical framework? Observed that The theoretical framework was poorly operationalized in the study.

   Response: We have revised the sentence in lines 106-107 page 7 (previously line 94) to make it clear that the factors we examined refer to cervical cancer screening awareness, structural barriers and psychosocial barriers.

7. Comment 7: METHODOLOGY: The cross-sectional study did not mention clearly the populations of the communities selected and sample size computation to indicate the appropriate sample size for such survey considering the implications of generalizability of the results obtained as the statements in the study concerning prevalence of the disease in the region.

   Response: This was an exploratory study and hence the sample size was driven by resource availability. It serves as a foundation for future studies on cervical cancer screening in this region. We used convenience sampling. In the Discussion, lines 322-329 page 17, we described the limitations of the study that we did not have access to population rosters and thus were unable to determine if participants were different from non-participants, and that the results may not be generalized to other areas. It is also possible that the lack of rural-urban differences observed in our study was due to the small sample size, and we have added this as a limitation in lines 324, page 17.

8. Comment 8: Line 133-134 may have introduced some bias Participants then watched short videos that described the cervical cancer screening methods available in Vietnam.

   Response: For clarification, participants completed the sociodemographic and the cervical cancer awareness questionnaires before watching the video. The participants then watched the videos explaining different screening approaches prior to completing the questionnaire on cervical cancer screening uptake and beliefs about the different types of screening. We believe it is important for participants to watch videos to provide consistent information about screening as women might not know what cervical cancer screening is and whether they had had it. In fact, many participants told the researchers that they realized that they had previously had cervical cancer screening after watching the videos. Therefore, we believe that asking participants to watch short videos describing the cervical cancer screening methods reduced misclassification rather than introducing bias. We provided the justification in lines 285-286 (page 15) and lines 321-322 (page 17). We have further provided the justification early in the Methods lines 148-149 page 9.

1415 Washington Heights
SPH Tower, Office
Ann Arbor, Michigan 48109-2029
phungmt@umich.edu

9. Comment 9: Data analysis needs to be adequately organized to mention all variables in the study and how these are measured. For example; cervical cancer screening uptake represents health-seeking at what time and what symptoms trigger desire to go for a test and how are these scored. The tables all have frequency distributions for every items in the instrument. This makes the result boring to read and confusing. The scores of the participants regarding moderating variables in the theoretical framework are absent even thought study claimed to have collected them.
   With the Likert scale weighted-aggregate score for each participant would have compressed all item to a single score for each participant and the mean with standard deviation synthesized. Statistical analysis in the study are grossly inadequate.

   Response: We did not collect the information on what symptoms triggering the desire to get screened because the purpose of the study is to promote regular cervical cancer screening even when women have no symptoms. As mentioned above, the purpose of the study is to assess the factors associated with cervical cancer screening (including awareness, structural and psychosocial barriers) to identify opportunities and challenges to improve screening in this population. We believe that presenting individual health belief items (as well as awareness and structural barriers) is easier to identify the opportunities and challenges to improve screening. In contrast, presenting an aggregate score compressing all items will obscure the items that need to be targeted to improve cervical cancer screening. Therefore, we would like to keep the way the data were presented. We are happy to move Table 4 to a supplemental table if the editor prefers it this way.

10. Comment 10: DISCUSSION: The discussion should not begin with the statement "..the first study to comprehensively examine cervical cancer…". what constitute comprehensive in the study? What makes it comprehensive when variables such as attitudinal dispositions of respondents, perceived benefits, self-efficacy expectations of taking a screening were not considered within the health belief construct.

    Response: We have revised the first sentence of the discussion as suggested (lines 280-281, page 15).

11. Comment 11: Line 265 "we found that ~50% of women reported ever being screened for cervical cancer…" contradict statement in line 36-37 "only a few Vietnamese women reported ever being screened for cervical cancer". About 50% cannot be regarded as few.

    Response: In line 39 (previously lines 36-37), we are referring to what was available in the literature prior to our study. We have revised this to make it clear.

12. Comment 12: CONCLUSION: To state in the conclusion in line 316 that lack of a national screening program in the study area contradicts "Screening timeframe recommended by Vietnam Ministry of Health" and other risk factors for the disease is a priority for intervention is inappropriate. Nowhere in the introduction did the author mention that national screening programme is a challenge. Health literacy was never a concern for the study, and why should this be part of the conclusion.

    Response: The Vietnam Ministry of Health has guidelines on the timeframe for cervical cancer screening. However, there is no national effort to promote public awareness of cervical cancer screening or to provide low- or no-cost screening. The latter point is particularly relevant given our

1415 Washington Heights
SPH Tower, Office
Ann Arbor, Michigan 48109-2029
phungmt@umich.edu

finding of cost being a barrier to screen. However, we have removed the language around a national screening program (lines 333-334, page 17). Health literacy is also relevant, as we found lack of awareness around cervical cancer screening.

Again, thank you for the opportunity to respond to the reviewers' comments.

Sincerely,

Minh Tung Phung, PhD, MPH

1415 Washington Heights
SPH Tower, Office
Ann Arbor, Michigan 48109-2029
phungmt@umich.edu
